# Death from Inhalation of Chloroform, in Edinburgh

**Published:** 1856-01

**Authors:** 


### ARTICLE XV.

### *Death from Inhalation of Chloroform, in Edinburgh.*

As Edinburgh has so long enjoyed an almost complete immunity from accidents resulting from the use of chloroform, the following case, which is reported in the *Edinburgh Medical Journal*, is worthy of attention. A lady, aged 36 years, called on Dr. W. A. Roberts, in order to have some teeth extracted. As she had inhaled chloroform once, during an accouchement, and as Dr. R. had also administered it to her on *four* previous occasions during the past year, he consented to employ it. She had only taken about nine or ten inspirations, when, in less than a minute from the time she began to inhale, *and while speaking*, she gave a convulsive start, and with a stertorous inspiration, and the eyes and mouth wide open, sunk to the floor. Dr. Simpson, being near at hand, was sent for, and arrived in less than five minutes, with Dr. Priestley. The means employed for relief, were artificial respiration, galvanism, and bleeding, though only a few ounces of blood could be obtained. After artificial respiration had been carried on for some time, spontaneous inspiration took place, the pulse became distinct, and the lividity of the face in a great measure disappeared. But these favorable indications ultimately declined, and after one hour and a quarter of the most energetic exertions (especially on the part of Dr. Simpson,) the case was reluctantly abandoned as hopeless, life being manifestly extinct.

At the *post-mortem* examination, the chief morbid appearances were found in the heart. This organ was very small, the right side flaccid and full of blood, the left firm and con tracted. The walls of the right side were unusually thin, and their tissue was soft and lacerable. Under the microscope, the muscular fibres of the right ventrical were much altered in appearance; the transverse striæ were indistinct, or had entirely disappeared in some portions, while fatty granules were everywhere observable, arranged in lines along the direction of the fibres.

The father of the patient had died of disease of the heart, being found dead in his chair.—*Bos. Med. & Sur. Jour.*

